# High Density Single Nucleotide Polymorphism (SNP) Mapping and Quantitative Trait Loci (QTL) Analysis in a Biparental Spring Triticale Population Localized Major and Minor Effect *Fusarium* Head Blight Resistance and Associated Traits QTL

**DOI:** 10.3390/genes9010019

**Published:** 2018-01-05

**Authors:** Raman Dhariwal, George Fedak, Yves Dion, Curtis Pozniak, André Laroche, François Eudes, Harpinder Singh Randhawa

**Affiliations:** 1 Agriculture and Agri-Food Canada, Lethbridge Research and Development Centre, Lethbridge, AB T1J 4B1, Canada; raman.dhariwal@agr.gc.ca (R.D.); andre.laroche@agr.gc.ca (A.L.); francois.eudes@agr.gc.ca (F.E.); 2 Agriculture and Agri-Food Canada, Ottawa Research and Development Centre, Ottawa, ON K1A 0C6, Canada; george.fedak@agr.gc.ca; 3 Centre de recherche sur les grains (CÉROM), Saint-Mathieu-de-Beloeil, QC J3G 0E2, Canada; yves.dion@cerom.qc.ca; 4 Crop Development Centre, University of Saskatchewan, Saskatoon, SK S7N 5A8, Canada; curtis.pozniak@usask.ca; 5 Department of Agricultural, Food & Nutritional Science, University of Alberta, Edmonton, AB T6G 2P5, Canada

**Keywords:** doubled haploid, *Fusarium* head blight, ergot, disease resistance, plant height, grain protein content, test weight, grain yield, epistasis

## Abstract

Triticale (x*Triticosecale* Wittmack) is an important feed crop which suffers severe yield, grade and end-use quality losses due to *Fusarium* head blight (FHB). Development of resistant triticale cultivars is hindered by lack of effective genetic resistance sources. To dissect FHB resistance, a doubled haploid spring triticale population produced from the cross TMP16315/AC Ultima using a microspore culture method, was phenotyped for FHB incidence, severity, visual rating index (VRI), deoxynivalenol (DON) and some associated traits (ergot, grain protein content, test weight, yield, plant height and lodging) followed by single nucleotide polymorphism (SNP) genotyping. A high-density map consisting of 5274 SNPs, mapped on all 21 chromosomes with a map density of 0.48 cM/SNP, was constructed. Together, 17 major quantitative trait loci were identified for FHB on chromosomes 1A, 2B, 3A, 4A, 4R, 5A, 5R and 6B; two of incidence loci (on 2B and 5R) also co-located with loci for severity and VRI, and two other loci of VRI (on 1A and 4R) with DON accumulation. Major and minor loci were also identified for all other traits in addition to many epistasis loci. This study provides new insight into the genetic basis of FHB resistance and their association with other traits in triticale.

## 1. Introduction

Triticale (x*Triticosecale* Wittmack; 2n = 6 × = 42) crop suffers severe losses in yield, grade and end-use quality due to *Fusarium* head blight (FHB) caused by *Fusarium graminearum*, *Fusarium avenaceum*, *Fusarium poae* and other spp. of genus *Fusarium* [1]. Where losses to kernel weight can be up to 30–70% [2], the contaminations by mycotoxins such as deoxynivalenol (DON), nivalenol and zearalenone, which are produced by *Fusarium* spp., are of foremost concern due to their harmful effects on human and animal health [3,4,5]. Growing resistant cultivars (cv.) is the most promising approach to manage FHB infections [6,7]. Three main modes of FHB resistance (Type-I: resistance to initial infection or disease incidence (DI), Type-II: resistance to spread of symptoms within the head or disease severity (DS) and Type-III: resistance to DON accumulation) along with three others (Type-IV: insensitivity to DON, Type-V: resistance to kernel infection or *Fusarium* damaged kernels (FDK) and Type-VI: tolerance or ability to produce marketable grains in the presence of FHB) have been reported in small grain cereals like wheat, barley and triticale [8,9]. Like wheat and barley, triticale cvs. predominantly possess quantitative resistance (mainly Type-II) with small and additive effects on FHB [1,10] and a high genotype and environment interaction [11,12] (reviewed elsewhere [13]).

Along with above complexities and quantitative nature of FHB resistance, its negative association with other traits complicates resistance evaluation and breeding. Recent studies reported a poor or negative correlation among different types of FHB resistances, particularly between DS and DON accumulation in wheat [14,15,16,17]. DS has also been found to be negatively associated with plant height (PHT) in wheat and triticale [3,4,5,18,19,20,21,22] because of co-segregation of FHB resistance loci with quantitative trait loci (QTL) for tallness [3,4,23,24]. Recent proteomics studies [25,26] on FHB resistance identified some general defense mechanism-related proteins/enzymes including α-amylase inhibitors [26] which were over-expressed in FHB inoculated wheat plants. The α-amylase inhibitors, also known as cereal seed allergens [27], are responsible for grain softness [28]. These reports indicate that there may be negative effects of increased α-amylase inhibitors on grain protein content, which is often high in hard grain varieties and low in soft grain varieties. Along with above findings, McCartney et al. [20] also found a significant negative correlation between FHB resistance (conferred by prominent Sumai-3 5AS QTL allele) and grain protein content in wheat, which states the necessity of identifying new resources of FHB resistance with minimized or no negative effects on other traits.

Amongst the small grain cereals (triticale, rye, oats, barley and wheat), triticale held fifth position in Canada in terms of area harvested (12,200 ha; total production: 33,000 metric tons) in 2017 [29] though it is mainly used for grazing, silage, ethanol production, and as grain for animal feed and a small proportion for human consumption market (reviewed elsewhere [13]). Even though triticale, particularly spring type, offers an excellent alternative to other small grain cereals for both feedstock and human consumption, it is still perceived to be a poor grain crop because of its susceptibility to ergot and FHB. Unlike the progress made in wheat, where a vast number of QTL identification studies for FHB resistance have been conducted [30] (for a review [31]), only three studies have been reported in triticale [3,4,5]. Besides the challenges related to nature of FHB resistance and their negative correlation with favorable agronomic characteristics, this obstacle can partly be attributed to unavailability of complete genome sequence and high-throughput marker system in triticale. Emerging resources of genome sequences (i.e., IWGSC Reference Sequence v1.0 and TGACv1 wheat genome assemblies recently generated by the International Wheat Genome Sequencing Consortium and Earlham Institute, respectively, are available for download and BLAST analysis) and high-throughput single nucleotide polymorphism (SNP) marker assays for wheat (i.e., 90K Infinium iSelect SNP Assay) and rye (i.e., rye 10K SNP Assay; KWS LOCHOW GMBH, Bergen, Germany), can accelerate the genotyping and genome wide studies including generation of accurate high density genetic maps in triticale. Keeping this in mind, we used wheat (90K) and rye (10K) SNP assays for genotyping a triticale doubled haploid (DH) population TMP16315/AC Ultima and constructed a high density SNP genetic map which was further utilized for mapping of additive, epistatic and QTL × environment interactions effects of *Fusarium* head blight (FHB) Type-I (DI), -II (DS), -III (DON), visual rating index (VRI) and ergot (ERG) resistances, grain protein content (GPC), test weight (TWT), grain yield (YLD), plant height (PHT) and lodging (LDG).

## 2. Materials and Methods 

### 2.1. Plant Material

A doubled haploid mapping population comprising of 100 lines was generated from a biparental cross between an uncharacterized spring triticale line TMP16315 and a Canadian triticale cv. AC Ultima [32] using the microspore culture method [33] at Lethbridge, AB. TMP16315 possess moderate FHB resistance, including DI (Type-I), DS (Type-II), VRI and DON accumulation (Type-III) along with moderate Ergot resistance, short height, low LDG and high TWT and YLD. AC Ultima is superior in GPC than TMP16315. Pronghorn (T124; spring triticale), Brevis (T200; spring triticale), FL62R1 (spring wheat), AC Barrie (spring wheat), Roblin (spring wheat) and Sumai3 (spring wheat) were used as controls/checks. 

### 2.2. FHB Inoculations and Phenotyping 

The DH lines and their parents as well as checks were screened in the FHB nurseries at Beloeil (BEL) and Ottawa (OTT) during 2011. Trial entries and checks were replicated 3 times in a randomized block design. Rows belonging to all entries were inoculated twice with mixture of macro-conidia of virulent isolates of *F. graminearum*. Visual observations at both locations were taken at 18 to 21 days post-inoculation. Percentage of heads infected (DI) and percentage of spikelets infected (DS) were measured and used for calculating the FHB VRI (DI × DS/100) [34]. Two 1 g aliquots were sampled from mature threshed heads from each plot followed by their grounding using a Romermill (Model 2A, Romer Labs Inc., Union, MO, USA) to extract DON. Bulked samples from the three replications of each line were used for quantification of DON using EZ-Quant^®^ Vomitoxin ELISA kit (Diagnostix, Ltd., Mississauga, ON, Canada) with an accuracy of 0.5 ppm [35].

### 2.3. Phenotyping of Other Traits

For the study of all other traits, all entries were planted (in a randomized block design) at Lethbridge (LET) location during 2011. Phenotypic observations for PHT (cm) and LDG (0–9 scale; 0 = erect, 9 = flat) [36] were taken at maturity for each line followed by harvesting of heads. Harvested heads were threshed and bulked samples from each line were used for measuring ERG incidence (pieces/Litre), % GPC (using whole seed, dry matter basis, by near infrared transmittance spectroscopy (Grainspec, Foss Food Technology, Brampton, ON, Canada)), TWT (kg/hL) and YLD (kg/ha). 

### 2.4. Phenotypic Data Evaluation

The phenotypic data was subjected to ANOVA using agricolae (version 1.2–4) package of R (R version 3.2.3) [37]. For the ANOVA model, DHs, their parents and checks were considered fixed effects, while environments and blocks were considered random effects. The ANOVA was conducted both within and across environments. Two combined ANOVA tables, one for all the FHB related traits and another for all other associated traits were generated. Pearson correlations and regression between traits and scatterplot were calculated for single environments using the R packages RCurl, caret, psych and functions cor.prob and flattenSquareMatrix (R version 3.2.3) [37]. 

### 2.5. DNA Isolation and Genotyping

For DNA isolation, two seeds from each of the DH line as well as parents were seeded in 96 cell seed planting trays in soil mixture of Turface (9.07 kg), Peat Moss (0.907 kg) and Vermiculite (0.06 cubic metre). Leaf tissue samples were collected 10 days post-seeding followed by DNA isolation using DNeasy 96 Plant Kit (Qiagen Inc., Valencia, CA, USA) following the manufacturer’s instructions. DNA was quantified using Quant-iT™ PicoGreen^®^ dsDNA Assay Kit (Thermo Fisher Scientific Inc., Bartlesville, OK, USA) and all DNA samples were diluted to 50 ng/µl. High-throughput SNP genotyping was carried out using wheat 90K Infinium iSelect SNP Assay and rye 10K SNP Assay (provided by KWS LOCHOW GMBH, Bergen, Germany). SNP genotyping data was analyzed using Genotyping module of GenomeStudio software package (Illumina Inc., San Diego, CA, USA). High quality SNPs were selected from the list of all SNPs evaluated for genotyping using following logical expression filter features in GenomeStudio (i) AA Freq: !=1, (ii) AB Freq: !=1, (iii) BB Freq: !=1, (iv) Minor Allele Freq: >0.03, and (v) Call Freq: >0.50. Monomorphic SNPs were removed from the list of selected SNPs by comparing their parental profile for identical SNP alleles. Selected SNPs that showed segregation distortion were removed using a ‘two steps’ strategy; in the first step, SNPs with allele frequencies <0.4 and >0.6 were removed followed by the second step, where SNPs that deviated significantly from 1:1 ratio on the bases of χ^2^ values, were removed. DH lines, which were dispersed as outliers during cluster analysis in GenomeStudio, were also removed from genotypic data. Only high quality polymorphic SNPs belonging to good DH lines (93), clearly segregating for 1:1 ratio, were used for genetic linkage mapping.

### 2.6. Construction of SNP Linkage Maps

High-quality SNP markers were used to construct linkage maps in two steps. In the first step, MSTMap software (version 2.0) [38]—available freely at GitHub—was used to construct draft maps. Following thresholds/parameters were used for computation: (i) Kosambi mapping function [39], (ii) no mapping size threshold of 3, (iii) no mapping distance threshold of 12.0 cM, (iv) no mapping missing threshold of 10%. In the second step, MapDisto software (version 1.7.7.011) [40] was used to refine linkage groups (LGs) generated in the previous step. Recombination (r) cut-off value 0.35 and LOD (logarithm of odds) threshold score 5.0 with Kosambi mapping function [39] were used to calculate distances (cM) between SNPs. Double recombinants were corrected after re-scoring. LGs were assigned to triticale chromosomes (chrs) based on the wheat 90K consensus SNP map [41], a rye reference map [42] and some unpublished data of rye and triticale (provided by Dr. Viktor Korzun, KWS LOCHOW GMBH, Bergen, Germany). Different LGs generated from the same homologue chr were merged to a single LG by using less stringent cut off values (LOD = 3.0, *r* = 0.4) in MapDisto. The commands “AutoCheckInversions” and “AutoRipple” were used to generate best order of markers. Marker’s positions were also searched and assigned against 9K consensus SNP map [43], International Triticeae Mapping Initiative (ITMI) reference map, rye and triticale SNP reference maps (provided by Dr. Viktor Korzun, KWS LOCHOW GMBH, Bergen, Germany) and wheat-survey sequences generated by the international wheat genome sequencing consortium (IWGSC; http://www.wheatgenome.org). For the latter, 90K iSelect SNP assay probe sequences were BLAST searched (with at least 99% identity and 100% query coverage) against the Chinese Spring (CS) survey sequence data using NCBI standalone blast program [44]. 

### 2.7. Quantitative Trait Loci (QTL) Mapping

Software packages QTL Cartographer (version 1.6) [45,46] and QGene (version 4.3.10) [47] were used for mapping main effect QTL (M-QTL) separately for each environment using composite interval mapping method with mapping function Kosambi [39] and regression method “forwards and backwards cofactor (*p* < 0.05)”. A LOD score of 2.5 was used to declare a putative QTL. One thousand permutations were used to calculate a threshold LOD score for declaring a significant QTL. QTLNetwork (version 2.0) [48] was used for two-locus analysis to identify additive (A), epistatic and QTL × environment interaction (A*E) effects of QTL. Linkage map diagrams illustrating QTL positions were drawn using MapChart software (version 2.32) [49]. A Circos diagram representing the epistatic interactions in a circular network plot was generated using the OmicCircos (version 1.14.0) package [50] in R version 3.2.3 [37].

## 3. Results

### 3.1. FHB Development and DON Accumulation 

**Figure 1:**
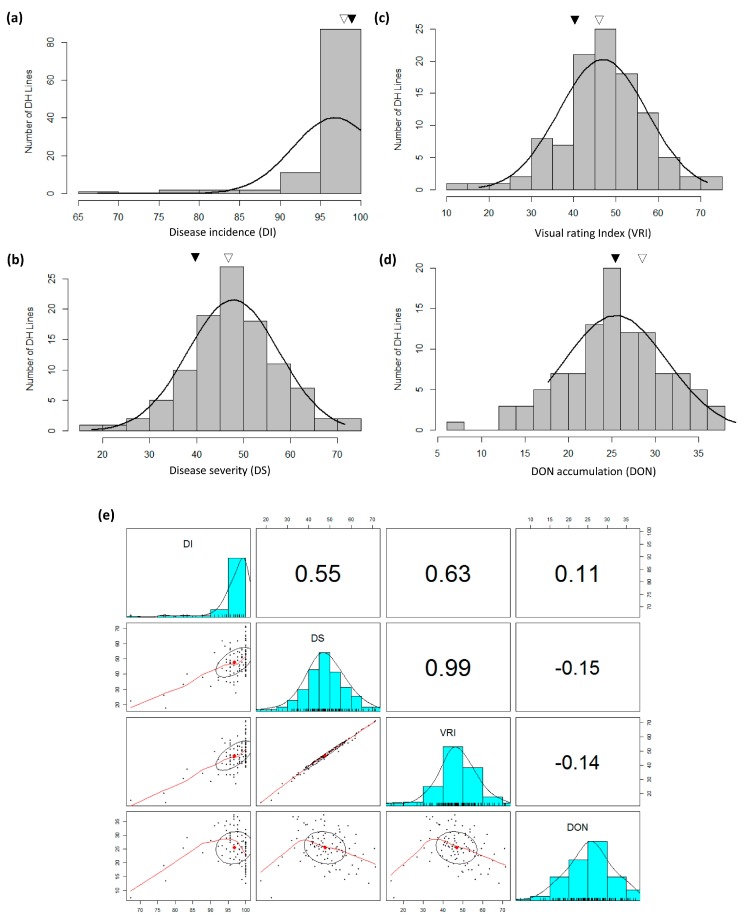
Frequency distribution of 100 doubled haploid (DH) lines of triticale genetic mapping population produced from a biparental cross between TMP16315 and AC Ultima for mean of four *Fusarium* head blight (FHB) traits namely: (**a**) disease incidence (DI, %), (**b**) disease severity (DS, %), (**c**) visual rating index (VRI, %) and (**d**) deoxynivalenol (DON, ppm) content evaluated at Beloeil. The means of the parental genotypes are indicated over graphs by triangles (AC Ultima, hollow triangle; TMP16316, solid triangle). (**e**) Scatterplot of above four FHB traits (DI, DS, VRI and DON) evaluated at Beloeil. Correlation coefficients (top diagonals) and regression line (lower diagonals) deciphered relationship among different traits studied.

**Figure 2:**
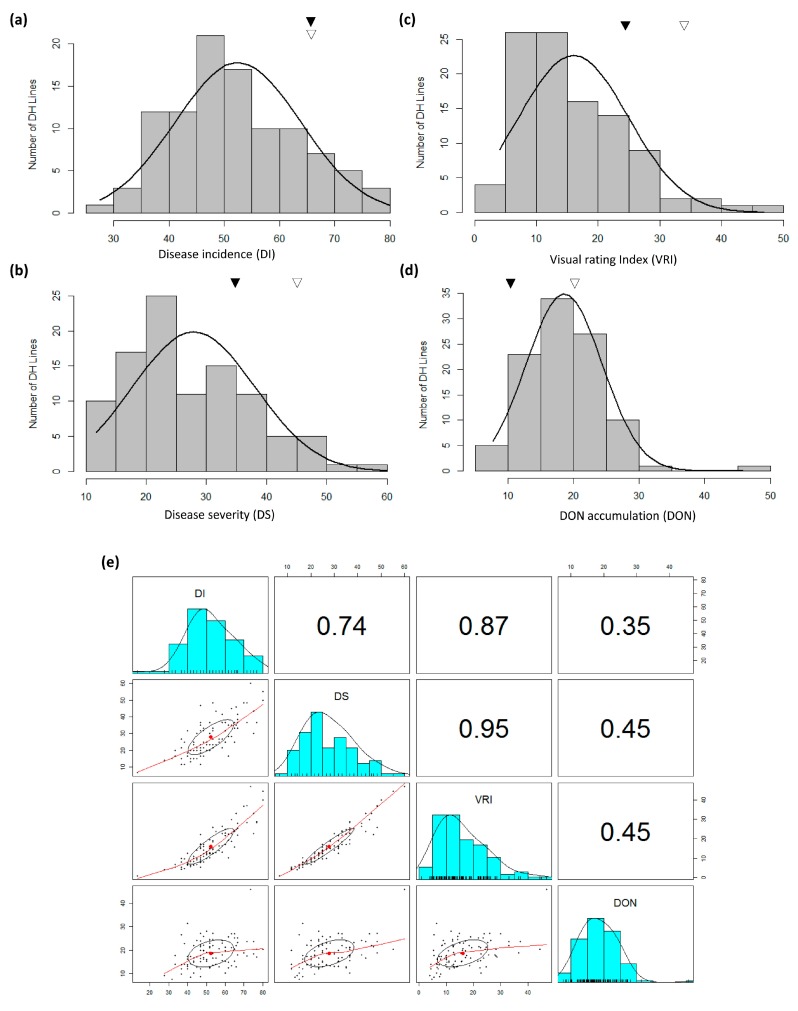
Frequency distribution of 100 doubled haploid lines of triticale genetic mapping population produced from a biparental cross between TMP16315 and AC Ultima for mean of four FHB traits namely: (**a**) (DI, %), (**b**) (DS, %), (**c**) (VRI, %) and (**d**) (DON, ppm) content evaluated at Ottawa. The means of the parental genotypes are indicated over graphs by triangles (AC Ultima, hollow triangle; TMP16316, solid triangle). (**e**) Scatterplot of above four FHB traits (DI, DS, VRI and DON) evaluated at Ottawa. Correlation coefficients (top diagonals) and regression line (lower diagonals) deciphered relationship among different traits studied.

Both FHB nurseries had good disease development with notably more FHB development at Beloeil (Supplementary Table S1). Among parents and common checks, while mean DI ranged from 7.00 to 65.80% at Ottawa, it was comparatively higher at Beloeil (ranged from 67.30 to 100.00%); almost 100.00% incidence was reported for both parents at Beloeil (Supplementary Table S1). Similarly, FHB DS and VRI were quite high at Beloeil (22.70 to 46.10% and 15.30 to 45.98%, respectively) in comparison to Ottawa (1.00 to 45.83% and 0.10 to 33.08%, respectively) but both parents showed much different phenotype at both locations. DON accumulation was also much higher at Beloeil (7.20 to 33.83%) in comparison to Ottawa (0.00 to 21.88%). Where both parents had indistinguishable DI in both environments, TMP16315 showed the lowest DS, VRI and DON. AC Ultima was more susceptible for DS, VRI and DON then TMP16315 or any other checks across both environments (Supplementary Table S1). At Beloeil, where population means for DS and VRI were above that of either parent, the population mean for DON was close to TMP16315. At Ottawa, population means were below the mean of either parent for DI, DS and VRI and close to the parental mean for DON (Supplementary Table S1). Population means for DI were below that of either parent at both locations. Phenotypic distribution of DS, VRI and DON were close to normal at Beloeil, whereas at Ottawa, only DI and DON had near normal distribution. A summary of means of different field disease reaction recorded on all checks across testing environments is presented in Supplementary Table S1 and histograms showing the distribution of DH lines are shown in Figure 1 and Figure 2. At Beloeil, downward transgressive segregants were observed for DI (Figure 1) and both upward and downward transgressive segregants for DS, VRI and DON; however, at Ottawa, largely downwards transgressive segregants were observed for DI, DS and VRI and both down and upwards for DON (Figure 2). At both locations, downward transgressive segregants indicate the presence of superior genes/QTLs for FHB resistance in both parents.

Correlation demonstrated a negative/weak relationship between FHB related traits (DI, DS and VRI), and DON accumulation at Beloeil (Figure 1) but a positive relationship at Ottawa (Figure 2) location. Regression analysis showed a very poor contribution (*r*^2^ = ~0.19 to ~0.25) of DI to DON accumulation at both locations (see far left-lower diagonal in Figure 1 and Figure 2) indicating the necessity to dissect the Type-III resistance (DON accumulation) separately by considering the environmental effect. Overall, the correlation analysis showed that where DI, DS and VRI are associated with each other (Figure 1 and Figure 2), their association with DON is largely influenced by environment. The ANOVA showed significant differences among the genotypes for all four traits in addition to significant environmental effect (Table 1).

### 3.2. Ergot Incidence, Test Weight, Grain Yield, Grain Protein Content, Plant Height and Lodging 

**Figure 3:**
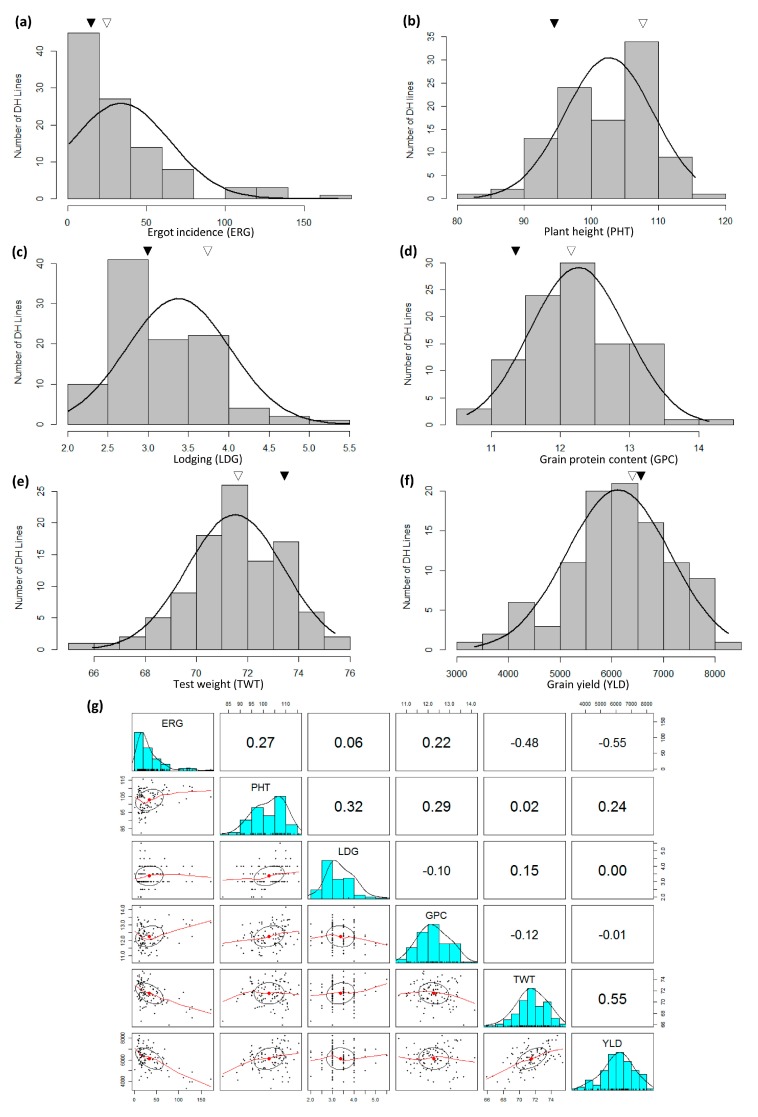
Frequency distribution of 100 doubled haploid lines of triticale genetic mapping population produced from a biparental cross between TMP16315 and AC Ultima for mean of six traits namely: (**a**) ergot incidence (ERG, pieces/L), (**b**) plant height (PHT, cm), (**c**) lodging (LDG, 1–9 scale), (**d**) grain protein content (GPC, %), (**e**) test weight (TWT, kg/hL) and (**f**) grain yield (YLD, kg/ha) evaluated at Lethbridge. The means of the parental genotypes are indicated over graphs by triangles (AC Ultima, hollow triangle; TMP16316, solid triangle). (**g**) Scatterplot of above six traits (ERG, PHT, LDG, GPC, TWT and YLD) evaluated at Lethbridge. Correlation coefficients (top diagonals) and regression line (lower diagonals) deciphered relationship among different traits studied.

Triticale genotype TMP16315 showed better phenotype (than AC Ultima) for ERG (low incidence), TWT (higher test weight), YLD (high), PHT (short) and LDG (low), while AC Ultima showed higher GPC (Supplementary Table S2). The DH lines showed significant variation for ERG, PHT, LDG, GPC, TWT and YLD (Figure 3; Supplementary Table S2). While skewed distribution was observed for ERG, near normal distributions (continuous variation) were observed for PHT, LDG, GPC, TWT and YLD. For ERG, though significant disease development was observed in parents, only upward transgressive segregation was observed where several DH lines showed higher ERG incidence than parents (Figure 3). Transgressive segregants in both directions were observed for PHT, LDG, GPC, TWT and YLD in the population (Figure 3).

Correlation analysis showed that while most of the above studied traits were not associated to each other or had very weak association (Figure 3); ERG had moderately negative association with TWT and YLD, both positively (though moderately) associated traits (Figure 3). ANOVA showed significant differences (*p* ≥0.05) among the genotypes for all the traits except GPC and yield but no replication/block or treatment × block interaction effect was observed (Table 2).

### 3.3. SNP Genetic Map

**Figure 4:**
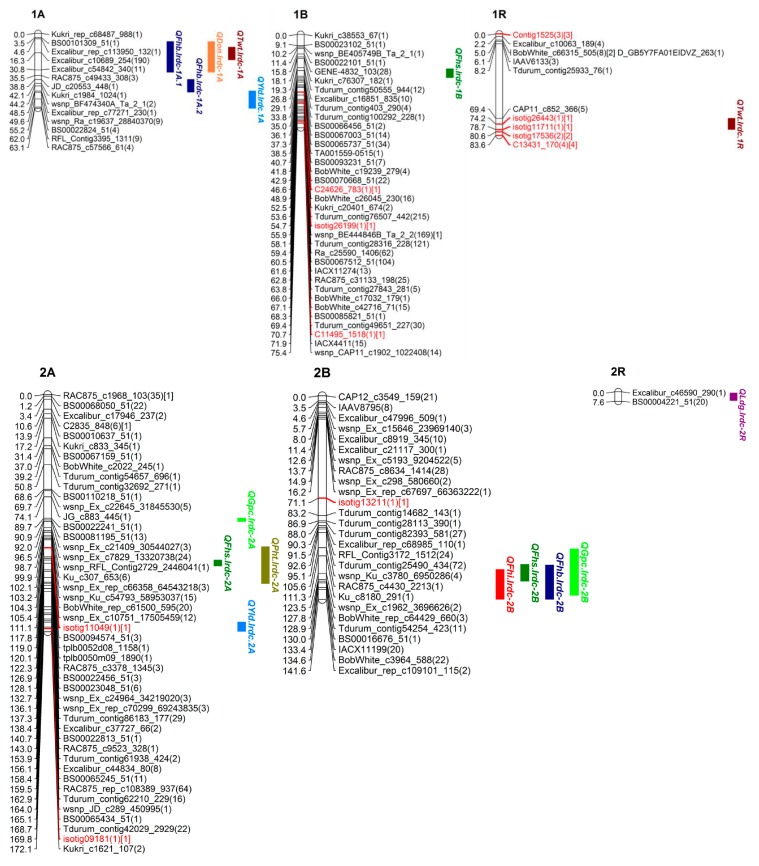
Homoeologous groups 1 and 2 single nucleotide polymorphism (SNP) map of TMP16315/AC Ultima triticale DH population. Loci on chromosome maps are represented by unique markers. Marker names in black and red colors represent genomic regions largely dominated by SNPs derived from wheat genomes A/B/D and rye genome R, respectively. Numbers in round brackets represents total number of SNPs at the locus and number in square brackets represents SNPs derived from R genome. The quantitative trait loci (QTL) for different traits are given on the right side of maps.

**Figure 5:**
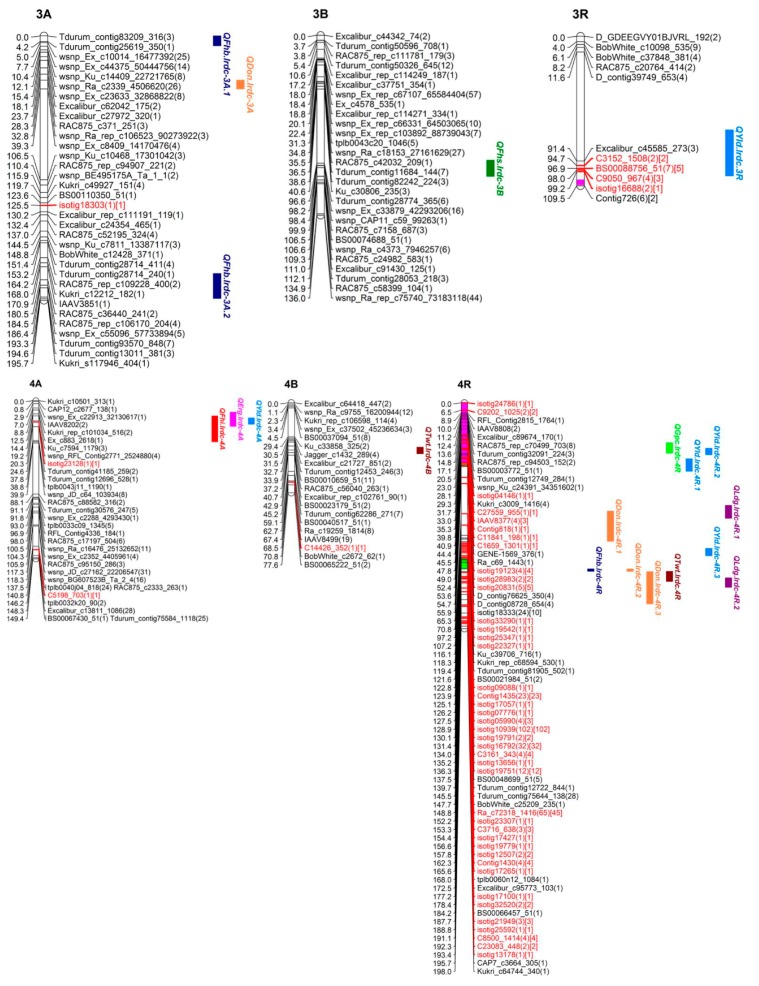
Homoeologous groups 3 and 4 SNP map of TMP16315/AC Ultima triticale DH population. Loci on chromosome maps are represented by unique markers. Marker names in black and red colors represent genomic regions largely dominated by SNPs derived from wheat genomes A/B/D and rye genome R, respectively. Numbers in round brackets represents total number of SNPs at the locus and number in square brackets represents SNPs derived from R genome. Colored chromosome bar indicates interchanged/translocated fragment (pink: chromosome (chr) 6R; green: chr 7R; brown: mosaic of chrs. 1, 2, 4, 5, 6 and 7). The QTL for different traits are given on the right side of maps.

**Figure 6:**
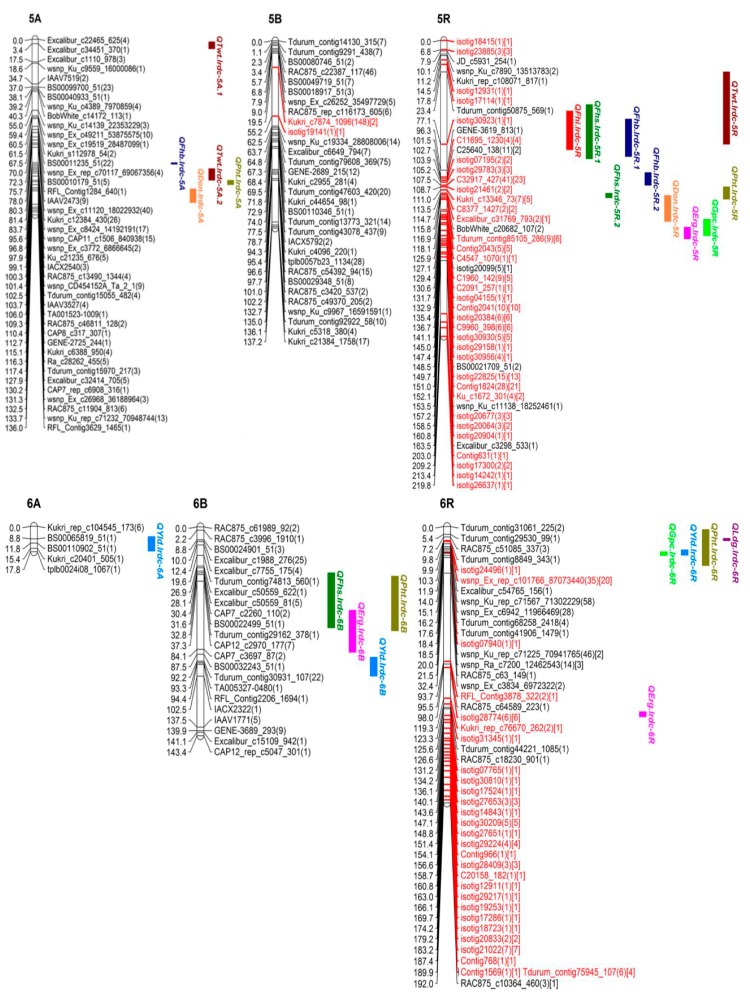
Homoeologous groups 5 and 6 SNP map of TMP16315/AC Ultima triticale DH population. Loci on chromosome maps are represented by unique markers. Marker names in black and red colors represent genomic regions largely dominated by SNPs derived from wheat genomes A/B/D and rye genome R, respectively. Numbers in round brackets represents total number of SNPs at the locus and number in square brackets represents SNPs derived from R genome. Red colored bar of chr 6R indicates interchanged/translocated fragment from chr 4R. The QTL for different traits are given on the right side of maps.

**Figure 7:**
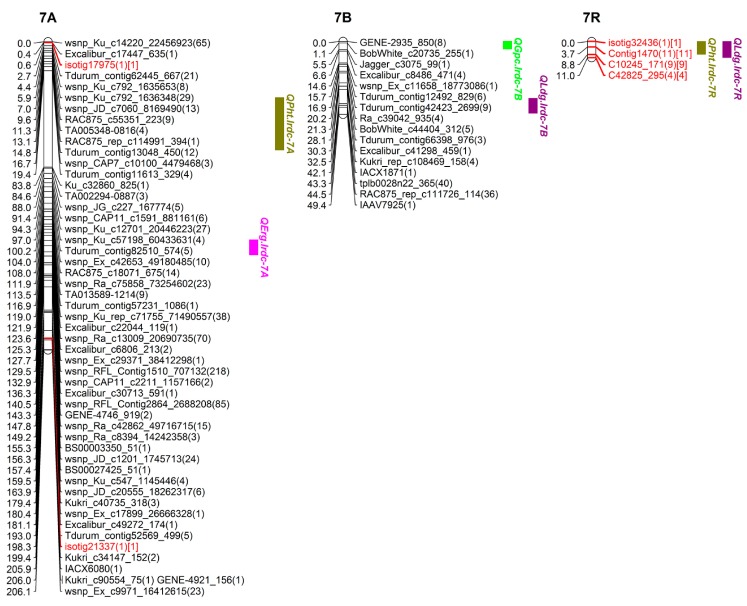
Homoeologous group 7 SNP map of TMP16315/AC Ultima triticale DH population. Loci on chromosome maps are represented by unique markers. Marker names in black and red colors represent genomic regions largely dominated by SNPs derived from wheat genomes A/B/D and rye genome R, respectively. Numbers in round brackets represents total number of SNPs at the locus and number in square brackets represents SNPs derived from R genome. The QTL for different traits are given on the right side of maps.

Out of over 100,000 SNPs (belonging to >90,000 from wheat 90K SNP Infinium iSelect assay and 10,000 from rye 10K SNP assay), a total of 5352 high quality polymorphic SNPs (4717 SNPs of wheat and 635 of rye) were selected for genetic mapping of triticale chrs. Around 99% SNPs (5274 out of total 5352; 4689 of wheat and 585 of rye) were genetically mapped to all 21 chrs (belonging to 3 homoeologous genomes namely A, B and R) in triticale population TMP16315 × AC Ultima (Figure 4, Figure 5, Figure 6 and Figure 7; Table 3). This map spanned a total of 2522.13 cM map distance (on average 120.10 cM per chr) with an average number of 2.09 SNPs per cM (Table 3). The highest number of SNPs were mapped to triticale homoeologous group 1 chrs (1226 SNPs; density = 5.52 SNPs/cM) and lowest number to group 6 chrs (367 SNPs; density = 1.04 SNPs/cM) but homoeologous group 3 showed lowest SNPs density (0.96 SNPs/cM) (Table 3). On the other hand, where genome B showed highest SNPs coverage (2249 SNPs; density = 2.96 SNPs/cM), which is similar to genome A (2021 SNPs; density = 2.15 SNPs/cM), genome R showed the lowest with almost half of the coverage (1004 SNPs; density = 1.22 SNPs/cM) of either A or B genome (Table 3). Despite the mapping of vast number of SNPs on triticale chrs and their high densities, the numbers of unique loci (all SNPs, that co-segregated among DH lines and mapped at same location on linkage map, were considered as single marker or unique locus) were 593, which spread over the total map length of 2522.13 cm. This led to an average density of one unique marker locus every 4.25 cM (ranging from 2.09 to 7.60) (Table 3). Genetic distance among unique loci also varied from 0.005 cM (on chr 6R; Supplementary Table S3) to 79.77 cM (on chr 3R; Figure 5 and Supplementary Table S3) which showed uneven distribution of loci along the chromosomal arms and the formation of clusters at certain regions; however, only 7.29% SNP interval exceeded 10.00 cM. A total of 1387 (out of total 5274) mapped SNPs in this study represent new mapped markers which were not present on either wheat consensus map [41] or previous triticale maps.

Additionally, a total of 0.42% SNPs (18 out of total 4270; 4270 includes 2021 and 2249 mapped on genomes A and B, respectively) mapped on genomes A and B together belonged to R genome (present on rye 10K SNP assay chip) and 43.52% (437 out of 1004) SNPs mapped on R genome of triticale belonged to wheat genomes A, B and D (present on wheat 90K SNP Infinium iSelect assay chip) (Table 3). We also observed chromosomal interchanges between chrs 4R and 6R in addition to translocations of small segments of wheat homoeologous group chrs 6 and 7 (or rye chrs 6R and 7R) to chrs 3R and 4R, respectively and a few segments of mosaic of wheat/rye chr groups 1, 2, 4, 5, 6 and 7 to chr 4R (Supplementary Table S3). The length of these interchanged/translocated segments varied from chr to chr (e.g., cluster of SNP loci co-localized (on chrs 3R, 4R and 6R), 8.00 cM (7R segment on 4R) and 20.82 cM (6R segment on 4R)). Some of these interchanged/translocated segments also carried some important genes/QTL co-segregating with different traits (i.e., FHB VRI, DON, LDG, GPC, TWT and YLD) (Tables 4, 6 and 7).

### 3.4. QTL Analysis

#### 3.4.1. QTL for FHB Resistance and Low DON Content

QTL analysis was carried out separately for DI, DS, VRI and DON which identified three main effect QTL for Type-I resistance [DI; on chrs 2B (*QFhi.lrdc-2B*), 4A (*QFhi.lrdc-4A*) and 5R (*QFhi.lrdc-5R*)], five main effect QTL for Type-II resistance [DS; on chrs 1B (*QFhs.lrdc-1B*), 2B (*QFhs.lrdc-2B*), 5R (*QFhs.lrdc-5R.1*, *QFhs.lrdc-5R.2*) and 6B (*QFhs.lrdc-6B*)], seven main effect QTL for VRI [on chrs 1A (*QFhb.lrdc-1A.1*), 2B (*QFhb.lrdc-2B*), 3A (*QFhb.lrdc-3A.1*), 4R (*QFhb.lrdc-4R*), 5A (*QFhb.lrdc-5A*) and 5R (*QFhb.lrdc-5R.1*, *QFhb.lrdc-5R.2*)] and seven main effect QTL for Type-III resistance [DON; on chrs 1A (*QDon.lrdc-1A*), 3A (*QDon.lrdc-3A*), 4R (*QDon.lrdc-4R.1*, *QDon.lrdc-4R.2*, *QDon.lrdc-4R.3*), 5A (*QDon.lrdc-5A*) and 5R (*QDon.lrdc-5R*)] (Figure 4, Figure 5 and Figure 6; Table 4). Seventeen of these QTL explained >10% of the phenotypic variation (*R*^2^) and were considered major QTL [51]. The total explained phenotypic variation ranged from 2.90 to 34.01% (Table 4). While all the DI and DS QTL were detected in both environments (Beloeil and Ottawa), only two QTL for VRI and one for DON were detected in both environments (Table 4); however, many other location specific QTL were detected for both, VRI and DON. Interestingly, two of the QTL regions on chrs 2B and 5R were commonly shared (co-localized/pleiotropic) among DI, DS and VRI, while two other QTL regions on chrs 1A and 4R were commonly shared between VRI and DON (Type-III resistance) (Table 4). These four QTL were considered major and the most promising QTL for FHB resistance and low DON content. On the other hand, ten of the identified QTL showed genotype × environment interactions (Table 4). The alleles for reduced FHB resistance (DI, DS, VRI and DON) were contributed by both parents; however, TMP16315 contributed more (14 out of total 22) favorable alleles, including alleles for 3 (out of all 4) commonly shared (co-localized/pleiotropic) QTL located on chrs 1A, 4R and 5R (Table 4).

**Figure 8:**
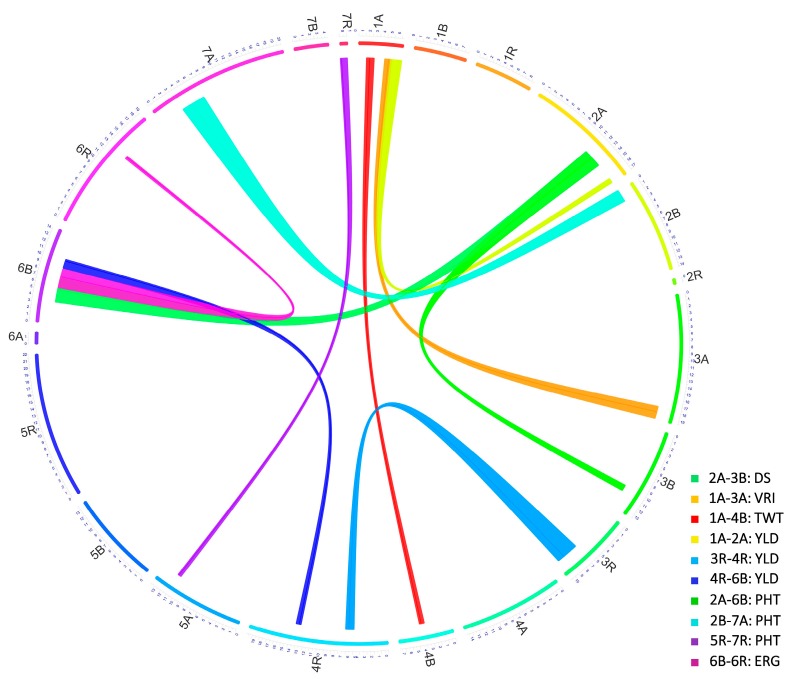
Epistasis QTL network. Circular plot represents the hexaploid triticale genome arranged in chromosomes (chrs) 1–21 (1A-7R) in clockwise direction in Circos diagram. Numbers on colored outer circle represents cM on respective chrs. Colored connections represent epistasis loci (QTL) controlling different traits (DS, VRI, TWT, YLD, PHT, ERG) and the widths of connections on respective chrs represent QTL intervals.

In addition to the above main effect QTL, epistasis QTL were also identified for DS and VRI on chrs 1A, 2A, 3A and 3B (Figure 8; Table 5). These epistasis QTL also showed significant additive effect and genotype × environment interactions. However, the alleles for reduced DS and VRI at epistasis QTL were contributed by AC Ultima (Table 5).

#### 3.4.2. QTL for Ergot Incidence

QTL analysis for ERG incidence identified three main effect QTL (located on chrs 4A (*QErg.lrdc-4A*), 5R (*QErg.lrdc-5R*) and 7A (*QErg.lrdc-7A*)) (Figure 5, Figure 6 and Figure 7; Table 6). All of the three-main effect QTL explained >10% (ranged from 11–34%) phenotypic variation (*R*^2^) and were considered major QTL (Table 6). Two of above 3 QTL for ERG incidence, each located on chrs 4A and 5R (Figure 5 and Figure 6; Table 6), were commonly shared with YLD and GPC, respectively. Interestingly, the alleles for reduced ERG incidence and increased YLD at shared loci on 4A and increased GPC at shared loci on 5R were contributed by AC Ultima while reduced ERG incidence allele at shared loci on 5R was contributed by TMP16315. 

In addition to above main effect QTL, epistasis QTL were also identified for reduced ERG incidence on chrs 6B and 6R (Figure 6 and Figure 8; Table 7) of AC Ultima. The allele for reduced ERG incidence on 6B was also shared with other QTL for reduced DS (Table 4) and PHT (Table 7). At both epistasis loci, favorable alleles were contributed by AC Ultima.

#### 3.4.3. QTL for Grain Protein Content 

QTL analysis for GPC identified six main effect QTL (located on chrs 2A (*QGpc.lrdc-2A*), 2B (*QGpc.lrdc-2B*), 4R (*QGpc.lrdc-4R*), 5R (*QGpc.lrdc-5R*), 6R (*QGpc.lrdc-6R*) and 7B (*QGpc.lrdc-7B*)) (Figure 4, Figure 5, Figure 6 and Figure 7; Table 6). Five of these QTL explained >10% (ranged from 6.1–39.0%) phenotypic variation (*R*^2^) and were considered major QTL (Table 6). QTL on 2B, 4R, 5R and 6R were shared with other traits (Table 6). Except loci on chr 2B, the alleles on all loci for increased GPC, including shared loci, were contributed by AC Ultima. No epistasis loci were identified for GPC.

#### 3.4.4. QTL for Test Weight 

QTL analysis for TWT identified five main effect QTL (located on chrs 1R (*QTwt.lrdc.1R*), 4R (*QTwt.lrdc.4R*), 5A (*QTwt.lrdc.5A.1*, *QTwt.lrdc.5A.2*) and 5R (*QTwt.lrdc.5R*)) (Figure 4, Figure 5 and Figure 6; Table 6). All these QTL explained >10% (ranged from 12.0–23.0%) phenotypic variation (*R*^2^) and were considered major QTL (Table 6). Interestingly, one loci located on chr 5R for increased TWT was commonly shared (along with other shared loci) with reduced FHB DI, DS and VRI and the favorable alleles for all common traits were contributed by TMP16315. In addition to above main effect QTL, epistasis QTL were also identified for increased TWT on chrs 1A and 4B (Figure 4, Figure 5 and Figure 8; Table 7) and similar to other QTL, favorable alleles were contributed by TMP16315.

#### 3.4.5. QTL for Grain Yield

QTL analysis for YLD identified four main effect QTL (located on chrs 4A (*QYld.lrdc-4A*), 4R (*QYld.lrdc-4R.2*), 6A (*QYld.lrdc-6A*) and 6R (*QYld.lrdc-6R*)) (Figure 5 and Figure 6; Table 6). All these QTL explained >10% (ranged from 12.0–15.0%) phenotypic variation (*R*^2^) and were considered major QTL (Table 6). Two of these QTL, one each located on chrs 4A and 4R, were commonly shared with ERG and GPC, respectively; another QTL located on 6R was commonly shared with GPC, PHT and LDG. Interestingly, all alleles at these loci for increased YLD were contributed by AC Ultima. In addition to above main effect QTL, epistasis QTL were also identified for increased YLD on chrs 1A, 2A, 3R, 4R and 6B (Figure 8; Table 7) but except for epistasis QTL at loci on chrs 3R and 4R, favorable alleles at all other loci were contributed by TMP16315.

#### 3.4.6. QTL for Plant Height and Lodging

QTL analysis was carried out separately for PHT and LDG which identified four main effect QTL for reduced PHT (on chrs 5A (*QPht.lrdc-5A*), 5R (*QPht.lrdc-5R*), 6R (*QPht.lrdc-6R*) and 7R (*QPht.lrdc-7R*)) (Figure 6 and Figure 7; Table 6) and six main effect QTL for low LDG (on chrs 2R (*QLdg.lrdc-2R*), 4R (*QLdg.lrdc-4R.1*, *QLdg.lrdc-4R.2*), 6R (*QLdg.lrdc-6R*), 7B (*QLdg.lrdc-7B*) and 7R (*QLdg.lrdc-7R*)) (Figure 4, Figure 5, Figure 6 and Figure 7; Table 6). Phenotypic variation (*R*^2^) explained by these QTL ranged from 6.0 to 38.0%. One of the above identified QTL on chr 5R (*QPht.lrdc-5R*) for reduced PHT was commonly shared with reduced Type-II and -III resistance and interestingly favorable allele for these, reduced PHT, DS and DON content, were contributed by TMP16315 (Table 4 and Table 6). Except two loci for low LDG, both on chr 4R, all other loci for short PHT and low LDG were contributed by TMP16315. 

Additionally, epistasis QTL were identified for PHT (on chrs 2A, 2B, 5A, 6B, 7A and 7R) but not for LDG (Figure 8; Table 7). Except at loci on chrs 5A and 7R, reduced height alleles for other epistasis QTL were contributed by AC Ultima (Table 7).

#### 3.4.7. Co-Localized or Pleiotropic QTL 

In addition to co-localized (pleiotropic) QTL listed above, fifteen more (of all identified) QTL were also co-localized (within 10 cM interval) with other QTL. One region on chr 1A, which harbored QTL for VRI (*QFhb.lrdc-1A*) and DON (*QDon.lrdc-1A*), also harbored QTL for TWT (*QTwt.lrdc-1A*) (Table 4). Similarly, the 2A region for DS (*QFhs.lrdc-2A*) harbored a QTL for PHT (*QPht.lrdc-2A*), a 2B region for DI (*QFhi.lrdc-2B*), DS (*QFhs.lrdc-2B*) and VRI (*QFhb.lrdc-2B*) harbored QTL for GPC (*QGpc.lrdc-2B*), a 4R region for VRI (*QFhb.lrdc-4R*) and DON (*QDon.lrdc-4R.2*, *QDon.lrdc-4R.3*) harbored QTL for TWT (*QTwt.lrdc.4R*) and LDG (*QLdg.lrdc-4R*), another 4R region for GPC (*QGpc.lrdc-4R*) harbored QTL for YLD (*QYld.lrdc-4R.2*), a 5A region for DON (*QDon.lrdc-5A*) harbored QTL for PHT (*QPht.lrdc-5A*), a 5R region for DI (*QFhi.lrdc-5R*), DS (*QFhs.lrdc-5R.1*) and VRI (*QFhb.lrdc-5R.1*) harbored QTL for TWT (*QTwt.lrdc-5R*), another 5R region for DON (*QDon.lrdc-5R*) and PHT (*QPht.lrdc-5R*) also harbored QTL for DS (*QFhs.lrdc-5R.2*) and a 6R region for GPC (*QGpc.lrdc-6R*) and YLD (*QYld.lrdc-6R*) harbored QTL for PHT (*QPht.lrdc-6R*) and LDG (*QLdg.lrdc-6R*) (Table 4, Table 5, Table 6 and Table 7).

## 4. Discussion

After the first report of *Fusarium* head blight (then scab) in England in 1884, many scientific studies have been conducted in wheat, barley and other cereals. The genetic factors, their chromosomal locations and sources (such as Sumai3, Wuhan, Nyubai and Frontana) for FHB resistance have been identified and well-studied in wheat [52]. However, progress in triticale is slow and only a few studies have been conducted [3,4,5]. Previous reports showed that FHB resistance in triticale is highly complex [53], predominantly quantitative in nature (mainly Type-II) with small additive effects [10] and possess a high degree of genotype × environment interaction [11,12]. In addition to above identified resistance elements, other factors such as existence of additive × additive (epistasis) interactions of resistance loci [54,55], different types (modes) of FHB resistance and poor correlation among them [7] and their association with unfavorable alleles of some agronomic characters such as tall plant type [4,5], makes FHB genetics more complex. Although a few attempts [3,4,5] have been made in the recent past to genetically dissect FHB resistance in winter triticale, no publicly available resources such as high-density SNP marker and QTL maps have been published yet for spring triticale. Thus, in the present study, a spring triticale DH population generated using the microspore culture method [33] was subjected to extensive phenotyping for FHB-DI, -DS, -VRI and DON and some other related traits (ERG, GPC, TWT, YLD, PHT and LDG) and to high-throughput genotyping (using >100,000 SNP marker belonging to wheat and rye genomes) which allowed construction of high density SNP and QTL maps for all the studied traits. Thus, the results of the present study add to our knowledge about the QTL that regulate FHB resistance and other related traits in spring triticale; more particularly, resources generated in this study, including the high-density SNP map and QTL for different traits, will facilitate rapid transfer of these loci into desirable lines using marker assisted breeding and will help in map based cloning. 

### 4.1. Phenotypic Data

Parents to make this DH population were selected for commercial breeding program and neither of the parents was highly susceptible for FHB, ERG and both parents shared many other favorable characters (Supplementary Tables S1 and S2). This increased the possibility of identification of FHB resistance (along with other traits) alleles from both parents and does not necessarily limit segregation variance [3,56]. LSD values showed that DH means for different traits either differed significantly and that a broad genetic variation existed (Supplementary Tables S1 and S2) or transgressive segregants provide new allele combinations which makes phenotypic data suitable for QTL mapping. The complete homozygosity of DH lines further enhances the QTL identification [4].

### 4.2. High Density Linkage Maps 

Gene associated SNPs, high-throughput genotype calling and low cost per data point of recently developed SNP assays for wheat (Infinium iSelect 90K SNP assay) [41] and rye (10K SNP assay; KWS LOCHOW GMBH, Bergen, Germany) opens new doors to accelerate the genotyping and genome wide studies in triticale. Out of the >100,000 SNPs used for the present study, a total of 5,274 (4689 from wheat and 585 from rye genome) markers representing 593 (451 from wheat and 142 from rye genome) unique loci were mapped on all 21 chrs of spring triticale. This showed that only ~5% of all utilized SNPs were informative for genetic mapping, which is expected as both parent did not differ much from each other and also showed high level of heterozygosity (and partly not discriminating between A/B/D and R genomes). This high level of heterozygosity is perhaps due to *Secale cereale* L. genome, which is a mosaic of various progenitor genomes [42]. Around 8.28% of total SNPs derived from A/B/D genomes were mapped on rye (R) genome; similarly, rye (R) genome SNPs (0.34% of total) were also mapped to A/B genomes (Table 3). Tyrka et al. [57] also observed cross mapping of R-genome markers on A/B genomes and A/B/D genome markers on R genome while working on Single Sequence Repeat (SSR), Diversity Array Technology (DArT) and DArTseq markers; however, where they observed 1.9% R genome markers on A/B genomes, only 0.9% of A/B/D genome markers were mapped to R genome (in comparison to 8.28% in our study). These results indicate the usefulness of 90K assay over SSR/DArT and other markers for linkage mapping in triticale. On the other hand, ~11.1% of total mapped SNPs represent R genome SNPs in this study, which is consistent with the ratio of number of A/B/D (wheat) genome markers (90K) to R (rye) genome markers (10K) tried for genetic mapping. The marker density of 4.24 cM/unique locus (0.48 cM/SNP) is comparable to recently published individual triticale maps, where it ranged from 3.0 cM/unique locus [57] to 5.2 cM/unique locus [58], however, none of these published maps used 90K Infinium iSelect SNP assay. Similarly, the genetic map length of 2522.13 cM also was comparable with other published maps of triticale, where individual map sizes ranged from 1745.0 [58] to 4907.0 [57] cM. On the other hand, individual genome size varied from genome to genome and A-genome had the longest map length followed by R and B genomes. Wang et al. [41] also observed A-genome as largest while working with wheat using 90K Infinium iSelect SNP assay. The SNP marker order for A and B genomes in this study was in very good agreement with wheat A and B genome maps [41] constructed previously using the 90K Infinium iSelect SNP assay and the marker order for R genome in this study was in very good agreement with previous R genome maps (rye and triticale SNP reference maps; provided by KWS LOCHOW GMBH, Bergen, Germany) or predicted first time using wheat survey sequences. The genetic map distances between loci varied from 0.005 cM (on chr 6R) to 79.77 cM (on chr 3R) and only 7.29% SNP interval exceeded 10cM. These results showed that the use of high-throughput SNP assays for genotyping in this study resulted in much improved genome coverage and several folds resolution compared to the previous individual DArT markers map [58]. We also observed uneven distribution of loci along the chromosomal arms and formation of large gaps and cluster at certain regions, which is in accordance with Alheit et al. [58] and can be explained by structural complexity and repetition of sequences found in cereal genomes. Perhaps, genomic complexities result in gaps and cluster of markers on genetic maps constructed using high-throughput SNP assays, since these assays mainly consisted of gene-based markers which represent only gene rich regions interspersed by significant number of repeat elements present in gaps. Proportions (26.3%; 1387/5274) of mapped SNPs in this study represent new mapped markers those were not present on either wheat consensus map [41] or previous triticale maps. These additional SNPs could mainly be result of addition of new germplasm, which was not present in Wang et al. [41] and use of additional rye SNPs for genotyping. This map provides enough coverage to dissect important traits segregating in this population.

### 4.3. Genetic Architecture of FHB Resistance in Triticale

Phenotypic data largely showed continuous variation for FHB-DI, -DS, -VRI and DON traits (Figure 1 and Figure 2). These observations agree with previous QTL analysis studies in triticale [3,4]. However, there are reports of poor or no association among different types of FHB resistance in wheat [14,15,16,17] and most of the previous triticale studies [3,4] concentrated only on DS, thus it is important to dissect other FHB resistance modes. Therefore, an extensive study involving DI, DS, VRI and DON was needed to dissect FHB resistance and identify common and unique QTL for different FHB resistance modes. Thus, in this study, QTL analysis was carried out separately for DI, DS, VRI and DON which enabled us to identify common and unique main effect and epistasis QTL for different modes of FHB resistance. These QTL, which mapped on all three genomes (A, B and R) of triticale included 3 main effect QTL for DI, 5 for DS, 7 for VRI and 7 for DON and 2 epistasis loci for DS and 2 others for VRI; many of these QTL were mapped finely to very short intervals (Figure 4, Figure 5, Figure 6, Figure 7 and Figure 8; Table 4 and Table 5). The individual QTL (main effect) explained up to 34% phenotypic variation (*R*^2^) (Table 4 and Table 5) which indicates their great potential for breeding programs. Many of the identified QTL for DI, VRI and DON on R genome in this study are merely reported in triticale (Table 4 and Table 5) though QTL identified on A and B genomes could have been located earlier in wheat. Kalih et al. [4] reported that all the wheat genomic regions co-localized with FHB resistance in their study in triticale were already mapped to wheat chrs in previous studies. The comparisons of positions of QTL identified in this study with all three previous studies in triticale [3,4,5] and most of the studies in wheat are difficult since different marker systems (DArT) were utilized earlier, while we used high-throughput SNP assays. In two recent wheat studies [59,60], 90K Infinium iSelect SNP assay was utilized but the QTL identified in these studies were not identified during the present study. In the present study, we identified some very important QTL regions for FHB resistance on chrs 1A (1 genomic region/pleotropic QTL; co-localized for VRI, DON and TWT), 2B (1; for DI, DS, VRI and GPC), 4R (1; for VRI, DON, TWT and LDG), 5A (1; for DON and PHT) and 5R (2; first for DI, DS, VRI and TWT and second for DS, VRI, DON and PHT), in addition to 5 (with >10% *R*^2^) another FHB/DON resistance QTL. Interestingly 1A QTL region (*QFhb.lrdc-1A.1*, *QDon.lrdc-1A*) was never identified in any of the previous triticale studies. This region also harbored an important QTL for TWT (*QTwt.lrdc-1A*; Table 7). Other QTL regions on chrs 2B, 4R and 5R may have already co-localized with DS previously using DArT marker system in winter triticale [4] but their association with other modes of resistance or other traits was detected for the first time in our study which may help in achieving high level of FHB resistance along with the simultaneous improvement of several traits through selection. QTL localized on chr 1A can also be helpful in achieving high level of FHB resistance with low DON and high-test weight since favorable allele for all these traits is derived from TMP16315. Similarly, other potential traits such as yield and ergot tolerance can also be improved simultaneously, since favorable allele donor is same (AC Ultima) (Table 6). QTL identified on chrs 2B, 4R and 5R are very prominent, with a significant additive effect and explained large phenotypic variations (Table 4 and Table 5). However, most of the QTL for FHB resistance (including DI, DS, VRI and DON) identified in this study had small to moderate additive effects (Table 4 and Table 5), except one QTL for VRI on chr 5A which had a large additive effect (additive effect: 12.0, LOD: 6.8 and *R*^2^: 29%) and was derived from AC Ultima, where most of the other important QTL were derived from TMP16315. This 5A QTL seems to be common with one of the three (3B, 5A and 6B) QTL of widely used Chinese cv. Sumai-3. However, phenotypic data indicate that AC Ultima possess low level of resistance in comparison to Sumai-3. This is obvious since AC Ultima may not have 3B and 6B QTL. However, effect of 5A QTL on FHB resistance can be validated in multiple environments for its further use in breeding programs. These observations suggest that while TMP16315 possess quantitative inheritance of minor additive genes/QTLs, AC Ultima carries a major QTL for FHB resistance along with some minor QTL. Since both parents, AC Ultima and TMP16315, contribute favorable QTL alleles, they can be utilized to pyramid and obtain a high level of FHB resistance in triticale. In this study, a few of the DH lines showed significantly better phenotype for FHB resistance than both parents. 

### 4.4. Genetic Architecture of Other Studied Traits and Their Possible Relation with FHB Resistance 

Previous studies showed negative relationship of FHB resistance with plant height [3,4,5,18,19,20,22], lodging [3], grain protein content [20] and yield [2] in wheat and/or triticale. Thus, to identify appropriate FHB resistance without a linkage drag, it was necessary to explore additional germplasm resources. In this study, phenotypic and QTL analysis was carried out separately for ERG, GPC, TWT, YLD, PHT and LDG. Phenotypic analysis showed that most of the above studied traits either had very weak association or were not associated to each other at phenotypic level except ERG tolerance which was moderately negatively associated with TWT and YLD, both positively associated traits (Figure 3). Therefore, QTL analysis was carried out to identify main effect and epistasis QTL as well as common shared/pleotropic QTL regions or QTL allele affecting other traits. Several main effect and epistasis QTL for all the traits from both parents were identified (Figure 4, Figure 5, Figure 6, Figure 7 and Figure 8; Table 6 and Table 7). These includes 3 main effect and 2 epistasis loci for ERG, 6 main effect loci for GPC, 5 main effect and 2 epistasis loci for TWT, 4 main effect and 6 epistasis loci for YLD, 4 main effect and 6 epistasis loci for PHT and 6 main effect loci for LDG (Table 6 and Table 7). Most of these QTL, particularly which are from rye genome (R), were reported for the first time except obviously some QTL e.g., YLD related QTL (A and B genome QTL mapped elsewhere during different wheat studies) and one for PHT (*QPht.lrdc-5R*); most likely this region was flanked by markers *Xiac130* and *Xiac128* in Kalih et al. [3] and/or *rPt509721* and *wPtm8731* in Kalih et al. [4]. However, only a single or few QTL from each trait showed large additive effect and rest of them showed minor additive effects such as *QErg.lrdc-7A* which had an additive effect of 34.0 and LOD score 8.1 (Table 6). Though, weak phenotypic association was observed for most of the traits, many of the identified QTL were also co-localized with other important genomic regions. Particularly, a QTL region on chr 4A of AC Ultima was found to be responsible for both reduced ergot incidence and increased grain yield (Table 6). Similarly, a chr 5R region of TMP16315, which carry QTL for low DI, DS and VRI, was responsible for high test weight. Another 5R region of TMP16315, which carry QTL for reduced DS, VRI and DON content, was also found responsible for short height. However, in case of latter QTL region (*QPht.lrdc-5R*) on chr 5R, which have also been most likely identified in earlier studies, Kalih et al. [3,4] observed negative association of reduced DS allele with PHT. During the present study, a common parental allele was identified to improve these traits simultaneously. This suggest that either tightly linked QTL for DS and PHT in this region or the QTL identified in previous studies are different than the loci identified during this study. On the other hand, a QTL allele from AC Ultima on a chr 2B region reduces FHB, another allele from TMP16315 reduces GPC. Similarly, a QTL allele from TMP16315 on a 5R region reduces ERG, another allele from AC Ultima increases GPC. Most likely these later results can be explained by the plant’s balance mechanism for growth and defense [61]. Since, growth-defense trade-offs are critical for crop breeding, it is recommended to use QTL/genes/homologs of existing known QTL/genes with no linkage drags in breeding programs.

## 5. Conclusions

The understanding of genetic architecture of FHB resistance and some related traits like ERG, GPC, TWT, YLD, PHT and LDG in spring triticale is important for developing superior cultivars. Therefore, for the first time, a high-density spring triticale SNP genetic map was generated and several new major and minor effects and epistasis QTL were mapped for FHB resistance and other related traits. These QTL includes both specific (such as a 3A QTL for reduced DON content) and common/pleotropic QTL (such as a 1A QTL for reduced VRI and DON and a 5R QTL for reduced DI, DS and VRI). On the other hand, both parents contributed favorable QTL alleles (such as 2B and 4A QTL for DI contributed by AC Ultima and TMP16315, respectively) and identified QTL expressed either across environments (such as all DI and DS related QTL) or specifically in an environment (such as 2B QTL for VRI and 1A QTL for DON). Most of the identified FHB resistance QTL were also co-localized with one or more agronomic traits such as a QTL on interstitial region of chr 5R for reduced DS, VRI and DON was found pleotropic for reduced PHT. At many pleotropic QTL regions, such as those mentioned above, the favorable alleles were contributed by same parent and these QTL can efficiently be used for marker-assisted selection (MAS) without any disadvantageous results to improve FHB resistance and other agronomic traits simultaneously. The high-density spring triticale SNP map also promises a starting platform for the fine mapping and map-based cloning of major stable QTL identified during the present study.

## Figures and Tables

**Table 1:** ANOVA of four FHB traits: DI; DS, VRI (= DI × DS/100) and DON (in ppm) of parents (TMP16315 and AC Ultima), their progenies (DHs) and checks, grown at Beloeil and Ottawa, during 2011.

Source	d.f.	DI	DS	VRI	DON
Environment (E)	1	306,278.0 **	55,591.0 **	136,890.0 **	7495.1 **
Block (within E)	2	680.0 **	119.0	177.0	278.2 *
Treatment (T)	104	439.0 **	496.0 **	474.0 **	148.7 **
(E × T)	100	212.0 **	205.0	201.0	70.9 **
Error	432	99.0	187.0	189.0	42.0
CV, %		13.5	36.7	44.7	29.5

CV: coefficient of variation; d.f.: degrees of freedom; Signif. codes: *p* ** ≤0.01, *p* * ≤0.05.

**Table 2:** ANOVA for ERG, PHT, LDG, GPC, TWT, YLD for parents (TMP16315 and AC Ultima), their progenies (DHs) and a check (Pronghorn), grown at Lethbridge (LET), during 2011.

Source	d.f.	ERG	PHT	LDG	GPC	TWT	YLD
Block (B)	1	290.9	103.9	0.8	0.4	1.4	2,624,730.0
Genotype (G)	100	1963.8 **	88.9 *	0.8 *	1.1	7.2 *	2,066,536.0
B × G	100	370.1	26.5	0.3	0.2	0.3	680,597.0
Error	6	144.2	27.7	0.2	0.9	1.2	1,327,087.0
CV, %		36.0	5.1	14.8	7.7	1.5	18.7

CV: coefficient of variation; d.f.: degrees of freedom; Signif. codes: *p* ** ≤0.01, *p* * ≤0.05.

**Table 3:** Details of number of mapped SNPs, map length and SNPs densities of individual chromosomes, seven homoeologous groups and three genomes (A, B and R genomes).

Genome and Chromosome	No. of SNPs (from R genome)	No. of Unique Loci (from R Genome)	Map Length (cM)	Marker Density		Map Density	
				SNPs/cM	Unique Loci/cM	cM/SNP	cM/Unique Locus
A 1	238 (0)	14 (0)	63.09	3.77	0.22	0.27	4.51
2	362 (4)	46 (4)	172.09	2.10	0.26	0.48	3.74
3	152 (1)	34 (1)	195.65	0.78	0.17	1.29	5.75
4	195 (2)	31 (2)	149.44	1.30	0.21	0.77	4.82
5	272 (0)	42 (0)	136.00	2.00	0.31	0.50	3.24
6	10 (0)	5 (0)	17.76	0.56	0.28	1.78	3.55
7	792 (2)	52 (2)	206.14	3.84	0.25	0.26	3.96
**Total**	2021 (9)	224 (9)	940.17	2.15	0.24	0.47	4.20
**%**	38.30 (0.17)	37.77 (1.52)					
B 1	955 (4)	36 (4)	75.40	12.67	0.48	0.08	2.09
2	274 (1)	27 (1)	141.59	1.94	0.19	0.52	5.24
3	225 (0)	28 (0)	135.99	1.65	0.20	0.60	4.86
4	94 (1)	20 (1)	77.58	1.21	0.26	0.83	3.88
5	479 (3)	31 (2)	137.16	3.49	0.23	0.29	4.42
6	97 (0)	22 (0)	143.39	0.68	0.15	1.48	6.52
7	125 (0)	16 (0)	49.36	2.53	0.32	0.39	3.09
**Total**	2249 (9)	180 (8)	760.48	2.96	0.24	0.34	4.22
**%**	42.60 (0.17)	30.35 (1.35)					
R 1	33 (13)	11 (6)	83.62	0.39	0.13	2.53	7.60
2	21 (0)	2 (0)	7.59	2.77	0.26	0.36	3.80
3	45 (13)	11 (5)	109.46	0.41	0.10	2.43	9.95
4	405 (290)	69 (42)	197.97	2.05	0.34	0.49	2.87
5	215 (147)	46 (37)	219.79	0.98	0.21	1.02	4.78
6	260 (79)	46 (31)	192.04	1.35	0.24	0.74	4.17
7	25 (25)	4 (4)	10.99	2.27	0.36	0.44	2.75
**Total**	1004 (567)	189 (125)	821.47	1.22	0.23	0.82	4.35
**%**	19.04 (10.80)	31.87 (20.08)					
A+B+R 1	1226 (17)	61 (10)	222.10	5.52	0.27	0.18	3.64
2	657 (5)	75 (5)	321.26	2.05	0.23	0.49	4.28
3	422 (14)	73 (6)	441.11	0.96	0.16	1.05	6.04
4	694 (293)	120 (45)	425.00	1.63	0.28	0.61	3.54
5	966 (150)	119 (39)	492.96	1.96	0.24	0.51	4.14
6	367 (79)	73 (31)	353.20	1.04	0.21	0.96	4.84
7	942 (27)	72 (6)	266.50	3.53	0.27	0.28	3.70
**Total**	5274 (585)	593 (142)	2522.13	2.09	0.23	0.48	4.25
**%**	100.00 (11.10)	100.00 (23.95)					

**Table 4:** Summary of FHB resistance related main effect QTL identified during the present study.

Trait	QTL Name	Chr.	Flanking Markers	Interval	Position	LOD	%*R*^2^	Add. Effect	A*E	D	Location	Other Associated Traits
DI	*QFhi.lrdc-2B*	2B	D_GDEEGVY01A58YG_63-RAC875_C6538_136	120.7–141.5	139.6	2.8	11.4	4.1	1.9	U	B, O	DS,VRI,GPC
(Type-I	*QFhi.lrdc-4A*	4A	TDURUM_CONTIG41185_259-TDURUM_CONTIG12696_528	14.4–38.8	33.6	3.1	14.1	−3.4	−1.5	T	B, O	-
Resistance)	*QFhi.lrdc-5R*	5R	TDURUM_CONTIG50875_569-GENE-3619_813	52.4–81.2	62.4	5.2	23.0	−5.8	−3.6	T	B, O	DS, VRI, TWT
DS	*QFhs.lrdc-1B*	1B	EXCALIBUR_C415_844-TDURUM_CONTIG100292_228	27.8–34.8	31.1	2.5	9.3	−2.7	−1.3	T	B, O	-
(Type-II	*QFhs.lrdc-2B*	2B	WSNP_EX_C1962_3696265-BOBWHITE_C2988_2161	117.2–128.8	123.4	2.7	11.4	3.5	1.3	U	B, O	DI, VRI, GPC
Resistance)	*QFhs.lrdc-5R.1*	5R	TDURUM_CONTIG50875_569-GENE-3619_813	47.7–87.9	77.0	3.7	20.6	−4.5	-	T	B, O	DI, VRI, TWT
	*QFhs.lrdc-5R.2*	5R	EXCALIBUR_C31769_793-BOBWHITE_C20682_107	113.5–116.8	114.7	3.2	15.1	−3.9	−1.6	T	B, O	VRI, DON, PHT
	*QFhs.lrdc-6B*	6B	TDURUM_CONTIG29162_378-CAP12_C2970_177	31.6–69.3	33.8	2.5	10.5	−3.2	−1.1	T	B, O	ERG, PHT
VRI	*QFhb.lrdc-1A.1*	1A	EXCALIBUR_REP_C113950_132-WSNP_KU_C3804_6986527	6.9–32.7	16.2	3.3	22.0	−0.5	-	T	B, O	DON, TWT
	*QFhb.lrdc-2B*	2B	D_GDEEGVY01A58YG_63-RAC875_C6538_136	117.7–141.5	127.8	7.7	32.0	3.8	-	U	O	DI, DS, GPC
	*QFhb.lrdc-3A.1*	3A	TDURUM_CONTIG25619_350-CAP12_C1860_280	0.0–7.3	4.2	3.3	4.9	2.2	-	U	B	-
	*QFhb.lrdc-4R* ^❀^	4R	IACX481-BOBWHITE_C25209_235	145.5–147.7	145.5	3.5	16.0	−3.7	−2.6	T	B	DON, LDG, TWT
	*QFhb.lrdc-5A*	5A	RAC875_C7132_134-EXCALIBUR_C92705_94	95.5–96.8	96.0	6.8	29.0	12.0	-	U	B	TWT
	*QFhb.lrdc-5R.1*	5R	TDURUM_CONTIG50875_569-GENE-3619_813	58.4–86.2	77.2	3.4	17.5	−3.5	1.7	T	B, O	DI, DS, TWT
	*QFhb.lrdc-5R.2*	5R	ISOTIG29606-C23413_289	98.1–107.5	103.9	5.9	26.0	−2.9	-	T	B	DS, DON, PHT
DON	*QDon.lrdc-1A*	1A	EXCALIBUR_REP_C113950_132-WSNP_KU_C3804_6986527	6.9–32.7	16.2	8.3	34.1	−3.0	-	T	O	VRI, TWT
(Type-III	*QDon.lrdc-3A*	3A	WSNP_CAP11_REP_C4157_1965583-WSNP_EX_C8409_14170476	32.8–39.3	34.1	5.9	26.0	2.4	-	U	O	-
Resistance)	*QDon.lrdc-4R.1*	4R	ISOTIG22327-KU_C39706_716	94.8–121.4	114.2	3.3	15.0	1.8	1.3	U	B, O	LDG
	*QDon.lrdc-4R.2* ^❀^	4R	IACX481-BOBWHITE_C25209_235	145.5–147.7	146.0	3.1	4.1	−1.2	-	T	B	VRI, LDG, TWT
	*QDon.lrdc-4R.3*	4R	ISOTIG23307-ISOTIG20098	147.9–176.2	152.3	3.0	2.9	−1.2	-	T	B	VRI, LDG, TWT
	*QDon.lrdc-5A*	5A	TDURUM_CONTIG15970_217-EXCALIBUR_C32414_705	115.9–126.5	119.3	6.9	30.0	2.7	-	U	B	PHT
	*QDon.lrdc-5R*	5R	TPLB0059H22_1233-CONTIG2043	115.1–134.5	119.6	3.3	5.6	−1.4	-	T	O	DS, VRI, PHT

Note: ^❀^ QTL located in interchanged fragment; LOD: logarithm of odds D: donor; T: TMP16315; U: AC Ultima; B: Beloeil; O: Ottawa.

**Table 5:** Summary of epistasis QTL for DS and VRI identified during the present study.

Trait	QTL Name	Chr.	Flanking Markers	Interval	Position	Add Effect	*p* Value	AA*E	*p* Value	D	Location	Other Associated Trait
DS (Type-II	*QFhs.lrdc-2A*	2A	BOBWHITE_C24021_254-BS00022456_51	120.1–124.3	122.3	0.9	0.000630	1.8	0.000054	U	B, O	PHT
Resistance)	*QFhs.lrdc-3B*	3B	RAC875_C7158_687-BS00074688_51	99.4–106.6	103.9							-
VRI	*QFhb.lrdc-1A*.2	1A	WSNP_BF474340A_TA_2_1-EXCALIBUR_REP_C77271_230	38.8–49.5	44.2	1.8	0.000013	−1.4	0.026233	U	B, O	-
	*QFhb.lrdc-3A.2*	3A	RAC875_REP_C106170_204-WSNP_EX_C55096_57733894	175.9–194.3	184.5							-

Note: D: donor; T: TMP16315; U: AC Ultima; B: Beloeil; O: Ottawa.

**Table 6:** Summary of main effect QTL for ERG, PHT, LDG, GPC, TWT and YLD identified during the present study.

Trait	QTL Name	Chr.	Flanking Markers	Interval	Position	LOD	%*R*^2^	Add Effect	A*E	D	Other Associated Traits
Ergot	*QErg.lrdc-4A*	4A	ISOTIG23128-TDURUM_CONTIG41185_259	10.8–24.3	20.3	2.5	11.0	8.9	6.2	U	YLD
	*QErg.lrdc-5R*	5R	ISOTIG30930-ISOTIG29158	138.7–147.4	143.1	2.6	12.0	−6.4	−3.9	T	GPC
	*QErg.lrdc-7A*	7A	EXCALIBUR_C30713_591-WSNP_EX_C5177_9174930	132.5–142.5	136.3	8.1	34.0	34.0	5.6	U	-
GPC	*QGpc.lrdc-2A*	2A	BS00022241_51-BS00081194_51	89.7–91.9	90.8	9.8	39.0	−0.3	-	U	-
	*QGpc.lrdc-2B*	2B	BOBWHITE_C2988_2161-BS00022572_51	106.5–138.6	127.8	2.5	6.1	0.1	0.1	T	DI, DS, VRI
	*QGpc.lrdc-4R* ^❀^	4R	C11841_198-C1659_1301	35.0–43.9	39.8	8.9	36.0	−0.7	−0.3	U	YLD
	*QGpc.lrdc-5R*	5R	ISOTIG30930-ISOTIG29158	132.9–145.0	141.1	9.0	37.0	−0.1	−0.2	U	ERG
	*QGpc.lrdc-6R*	6R	TDURUM_CONTIG41906_1479-WSNP_EX_C7002_12063380	17.6–20.0	19.9	3.0	17.8	−0.5	-	U	PHT, LDG, YLD
	*QGpc.lrdc-7B*	7B	EXCALIBUR_C3489_182-BOBWHITE_C20735_255	0.0–5.1	0.0	6.0	26.0	−0.2	−0.2	U	-
TWT	*QTwt.lrdc.1R*	1R	RAC875_REP_C116934_270-ISOTIG11711	69.4–78.6	69.5	3.1	15.0	0.5	-	T	-
	*QTwt.lrdc.4R* ^❀^	4R	BOBWHITE_C25209_235-ISOTIG19779	147.7–156.6	152.1	4.5	21.0	0.6	-	T	VRI, DON, LDG
	*QTwt.lrdc-5A.1*	5A	EXCALIBUR_C34451_370-KUKRI_C9358_269	2.0–7.4	3.4	4.7	21.5	−0.6	−0.9	U	-
	*QTwt.lrdc-5A.2*	5A	RAC875_C60453_122-WSNP_EX_C31154_39982416	100.2–109.2	101.4	5.3	23.0	0.7	-	T	VRI
	*QTwt.lrdc-5R*	5R	TDURUM_CONTIG50875_569-GENE-3619_813	23.4–77.1	50.1	2.5	12.0	0.6	-	T	DI, DS, VRI
YLD	*QYld.lrdc-4A*	4A	ISOTIG23128-TDURUM_CONTIG41185_259	16.4–22.3	20.3	2.9	14.1	−342.0	−128.1	U	ERG
	*QYld.lrdc-4R.2* ^❀^	4R	C1659_1301-GENE-1569_376	39.8–45.4	41.9	3.1	15.0	−97.6	−203.3	U	GPC
	*QYld.lrdc-6A*	6A	BS00110902_51-KUKRI_C20401_505	7.2–17.3	11.8	2.7	13.0	−358.4	-	U	-
	*QYld.lrdc-6R*	6R	TDURUM_CONTIG41906_1479-WSNP_EX_C7002_12063380	16.2–19.9	17.6	2.5	12.0	−371.0	−251.1	U	GPC, PHT, LDG
PHT	*QPht.lrdc-5A*	5A	CAP8_C317_307-GENE-2725_244	109.3–112.7	112.4	2.7	13.0	−1.9	2.2	T	DON
	*QPht.lrdc-5R*	5R	TPLB0059H22_1233-CONTIG2043	108.7–117.9	116.9	2.7	13.0	−2.4	−2.3	T	DS, VRI, DON
	*QPht.lrdc-6R*	6R	KUKRI_C9940_659-TDURUM_CONTIG41906_1479	2.1–27.1	16.3	9.4	38.0	−3.2	-	T	GPC, LDG, YLD
	*QPht.lrdc-7R*	7R	ISOTIG32436-ISOTIG07139	0.0–8.7	1.0	2.5	11.0	−1.0	−1.7	T	LDG
LDG	*QLdg.lrdc-2R*	2R	EXCALIBUR_C46590_290-BS00061187_51	1.0–7.0	7.0	2.5	11.7	−0.1	−0.1	T	-
	*QLdg.lrdc-4R.1*	4R	ISOTIG25347-ISOTIG22327	89.8–101.2	97.2	6.0	26.0	0.3	0.1	U	DON
	*QLdg.lrdc-4R.2*	4R	ISOTIG17427-ISOTIG19779	153.3–161.7	156.4	5.9	26.0	0.1	0.1	U	VRI, DON, TWT
	*QLdg.lrdc-6R* ^❀^	6R	TDURUM_CONTIG8849_343-ISOTIG24496	8.2–9.9	9.8	2.9	14.1	−0.14	−0.1	T	GPC, PHT, YLD
	*QLdg.lrdc-7B*	7B	WSNP_KU_C10355_17149304-IBV7925	38.5–48.5	44.5	2.5	6.0	−0.1	−0.1	T	-
	*QLdg.lrdc-7R*	7R	C10245_171-C6434_652	0.0–10.8	8.8	3.0	14.1	−0.2	-	T	PHT

Note: ^❀^ QTL located in interchanged fragment; D: donor; T: TMP16315; U: AC Ultima.

**Table 7:** Summary of epistasis QTL for ERG, PHT, TWT and YLD identified during the present study.

Trait	QTL Name	Chr.	Flanking Markers	Interval	Position	Add Effect	*p* Value	AA*E	*p* Value	D	Other Associated Traits
ERG	*QErg.lrdc-6B*	6B	CAP12_C2970_177-CAP7_C3697_87	57.3–86.1	74.3	3.3	0.002321	6.0	0.000859	U	DS, PHT
	*QErg.lrdc-6R*	6R	ISOTIG07765-ISOTIG30810	128.6–132.2	131.2						-
TWT	*QTwt.lrdc-1A*	1A	RAC875_C89908_105-WSNP_KU_C3804_6986527	11.6–22.3	16.3	0.6	0.015108	0.9	0.029488	T	DON, VRI
	*QTwt.lrdc-4B*	4B	EXCALIBUR_REP_C102761_90-BS00023179_51	38.2–43.9	41.7						-
YLD	*QYld.lrdc.1A*	1A	WSNP_EX_C18499_27344859-BS00070695_51	48.5–63.0	60.2	62.2	0.043176	102.1	0.026707	T	-
	*QYld.lrdc.2A*	2A	ISOTIG09181-EXCALIBUR_REP_C102984_157	165.1–171.8	170.8						-
	*QYld.lrdc.3R*	3R	IACX7129-C3152_1508	68.6–102.2	92.4	−108.9	0.000227	−219.0	0.000010	U	-
	*QYld.lrdc.4R.1* ^❀^	4R	TDURUM_CONTIG42642_656-ISOTIG33290	49.0–59.9	55.9						-
	*QYld.lrdc-4R.3*	4R	ISOTIG29781-ISOTIG09995	127.6–134.0	129.9	85.1	0.005414	159.4	0.001273	T	-
	*QYld.lrdc-6B*	6B	RFL_CONTIG2206_1694-IACX2322	89.5–102.4	100.4						-
PHT	*QPht.lrdc-2A*	2A	EXCALIBUR_REP_C111191_119-EXCALIBUR_C24354_465	110.4–137.0	131.2	1.5	0.001168	3.0	0.000160	U	DS
	*QPht.lrdc-6B*	6B	CAP12_C2970_177-CAP7_C3697_87	33.8–71.3	53.3						ERG, DS
	*QPht.lrdc-2B*	2B	EXCALIBUR_REP_C114249_187-EXCALIBUR_C37751_354	0.0–18.4	14.6	2.2	0.001360	4.1	0.000319	U	-
	*QPht.lrdc-7A*	7A	CAP11_C3214_133-KU_C32860_825	37.4–72.4	53.4						-
	*QPht.lrdc-5A*	5A	CAP8_C317_307-GENE-2725_244	109.3–112.7	112.4	0.9	0.013789	1.4	0.012962	T	DON
	*QPht.lrdc-7R*	7R	ISOTIG32436-ISOTIG07139	0.0–8.7	1.0						LDG

Note: ^❀^ QTL located in interchanged fragment; D: donor; T: TMP16315; U: AC Ultima.

## Supplementary Materials

The following are available online at www.mdpi.com/2073-4425/9/1/19/s1. Table S1: Details of phenotypic performance [for *Fusarium* head blight (FHB) related traits: disease incidence (percent heads infected; DI), disease severity (percent spikelets infected; DS), visual rating index (DI × DS/100 = VRI) and deoxynivalenol content (DON; in ppm)] of TMP16315 and AC Ultima, their progenies (doubled haploids; DHs) and checks used during the present study at Beloeil (BEL) and Ottawa (OTT) locations/environments. Table S2: Details of phenotypic performance [for ergot incidence (ERG), plant height (PHT), lodging (LDG), grain protein content (GPC), test weight (TWT) and grain yield (YLD)] of parents (TMP16315 and AC Ultima), their progenies (doubled haploids; DHs) and a check used during the present study at Lethbridge (LET) location. Table S3: Details of TMP16315/AC Ultima SNP genetic map.
