# Supplementary material for: High Density Single Nucleotide Polymorphism (SNP) Mapping and Quantitative Trait Loci (QTL) Analysis in a Biparental Spring Triticale Population Localized Major and Minor Effect Fusarium Head Blight Resistance and Associated Traits QTL
Source: Genes (Basel). 2018 Jan 5;9(1):19. doi: 10.3390/genes9010019 (PMC5793172; doi:10.3390/genes9010019)
Supplement: Supplementary file 1 [file genes-09-00019-s001.zip › Supplementary Tables S1 and S2.docx]

**Supplementary Table 1:** Details of phenotypic performance [for fusarium head blight (FHB) related traits: disease incidence (percent heads infected; DI), disease severity (percent spikelets infected; DS), visual rating index (DI × DS/100 = VRI) and deoxynivalenol content (DON; in ppm)] of TMP16315 and AC Ultima, their progenies (doubled haploids; DHs) and checks used during the present study at Beloeil (BEL) and Ottawa (OTT) locations/environments.

| Trait and Env | Parental lines | | | DHs | | | Common checks | | Other Checks | | | | LSD | %CV |
| --- | --- | --- | --- | --- | --- | --- | --- | --- | --- | --- | --- | --- | --- | --- |
|  | TMP16315 | AC Ultima | | Min | Max | Mean | Pronghorn (T124) | Sumai3 | Brevis  (T200) | FL62R1 | AC Barrie | Roblin |  |  |
| FHB DI (Type-I resistance) | | | | | | | | | |  |  |  |  |  |
| BEL | 100.0 | | 99.3 | 50.0 | 100.0 | 97.3 | 97.8 | 67.3 | 97.2 | 77.2 | 76.7 | - | 2.7 | 13.5 |
| OTT | 65.8 | | 65.8 | 30.0 | 95.0 | 52.5 | 27.5 | 7.0 | - | - | - | 50.0 | 3.2 |  |
| M | 82.9 | | 82.6 | 40.0 | 97.5 | 74.9 | 62.6 | 37.1 | - | - | - | - |  |  |
| FHB DS (Type-II resistance) | | | | | | | | | |  |  |  |  |  |
| BEL | 42.7 | | 46.1 | 9.9 | 88.7 | 48.9 | 38.8 | 22.7 | 27.8 | 17.8 | 26.5 | - | 6.6 | 36.7 |
| OTT | 35.0 | | 45.8 | 10.0 | 85.0 | 27.8 | 14.2 | 1.0 | - | - | - | 39.4 | 2.9 |  |
| M | 38.9 | | 46.0 | 9.9 | 86.8 | 38.3 | 26.5 | 11.8 | - | - | - | - |  |  |
| FHB VRI | | | | | | | | | |  |  |  |  |  |
| BEL | 42.7 | | 46.0 | 5.6 | 88.7 | 48.0 | 38.4 | 15.3 | 27.0 | 13.7 | 20.3 | - | 6.8 | 44.7 |
| OTT | 24.7 | | 33.1 | 3.0 | 80.8 | 15.9 | 4.0 | 0.1 | - | - | - | 20.0 | 2.8 |  |
| M | 33.7 | | 39.5 | 4.3 | 84.7 | 31.9 | 21.2 | 7.7 | - | - | - | - |  |  |
| DON (Type-III resistance) | | | | | | | | | |  |  |  |  |  |
| BEL | 26.0 | | 28.1 | 8.3 | 49.4 | 25.8 | 33.8 | 7.2 | 22.0 | 13.4 | 0.1 | - | 1.7 | 29.5 |
| OTT | 12.8 | | 21.9 | 3.0 | 52.7 | 18.9 | 9.2 | 0.0 | - | - | - | 11.9 | 1.9 |  |
| M | 19.4 | | 24.9 | 5.6 | 51.1 | 22.3 | 21.5 | 3.6 | - | - | - | - |  |  |

Note: M: mean of two locations; - : respective check was not used for the given location or mean was not calculated due to only one location.

**Supplementary Table 2:** Details of phenotypic performance [for ergot incidence (ERG), plant height (PHT), lodging (LDG), grain protein content (GPC), test weight (TWT) and grain yield (YLD)] of parents (TMP16315 and AC Ultima), their progenies (doubled haploids; DHs) and a check used during the present study at Lethbridge (LET) location.

| Trait | Parental lines | | DHs | | | Common  check | LSD | %CV |
| --- | --- | --- | --- | --- | --- | --- | --- | --- |
|  | TMP16315 | AC Ultima | Min | Max | Mean | Pronghorn (T124) |  |  |
| ERG (pieces/L) | 18.7 | 21.7 | 0.0 | 200.0 | 34.4 | 10.7 | 4.1 | 36.0 |
| GPC (%) | 11.3 | 12.2 | 10.1 | 14.3 | 12.3 | 10.6 | 0.3 | 7.8 |
| TWT (kg/hL) | 73.3 | 71.7 | 65.6 | 75.7 | 71.5 | 71.1 | 0.4 | 1.5 |
| YLD (kg/ha) | 6526.2 | 6427.3 | 3131.3 | 8792.6 | 6098.2 | 7983.8 | 390.9 | 18.7 |
| PHT (cm) | 95.5 | 107.7 | 82.0 | 117.0 | 102.6 | 106.2 | 1.8 | 5.1 |
| LDG (1-9) | 3.0 | 3.7 | 2.0 | 6.0 | 3.4 | 4.0 | 0.2 | 14.8 |
